# Strategies to combat the problem of yam anthracnose disease: Status and prospects

**DOI:** 10.1111/mpp.13107

**Published:** 2021-07-17

**Authors:** Valentine Otang Ntui, Edak Aniedi Uyoh, Effiom Eyo Ita, Aniedi‐Abasi Akpan Markson, Jaindra Nath Tripathi, Nkese Ime Okon, Mfon Okon Akpan, Julius Oyohosuho Phillip, Ebiamadon Andi Brisibe, Ene‐Obong Effiom Ene‐Obong, Leena Tripathi

**Affiliations:** ^1^ Department of Genetics and Biotechnology University of Calabar Calabar Nigeria; ^2^ International Institute of Tropical Agriculture Nairobi Kenya; ^3^ Department of Plant and Ecological Studies University of Calabar Calabar Nigeria

**Keywords:** anthracnose, CRISPR/Cas, *Dioscorea* spp., fungal diseases, genomics‐assisted breeding, new breeding techniques, RNAi, yam

## Abstract

Yam (*Dioscorea* spp.) anthracnose, caused by *Colletotrichum alatae*, is the most devastating fungal disease of yam in West Africa, leading to 50%–90% of tuber yield losses in severe cases. In some instances, plants die without producing any tubers or each shoot may produce several small tubers before it dies if the disease strikes early. *C. alatae* affects all parts of the yam plant at all stages of development, including leaves, stems, tubers, and seeds of yams, and it is highly prevalent in the yam belt region and other yam‐producing countries in the world. Traditional methods adopted by farmers to control the disease have not been very successful. Fungicides have also failed to provide long‐lasting control. Although conventional breeding and genomics‐assisted breeding have been used to develop some level of resistance to anthracnose in *Dioscorea alata*, the appearance of new and more virulent strains makes the development of improved varieties with broad‐spectrum and durable resistance critical. These shortcomings, coupled with interspecific incompatibility, dioecy, polyploidy, poor flowering, and the long breeding cycle of the crop, have prompted researchers to explore biotechnological techniques to complement conventional breeding to speed up crop improvement. Modern biotechnological tools have the potential of producing fungus‐resistant cultivars, thereby bypassing the natural bottlenecks of traditional breeding. This article reviews the existing biotechnological strategies and proposes several approaches that could be adopted to develop anthracnose‐resistant yam varieties for improved food security in West Africa.

## Graphical abstract

Resistance to yam anthracnose, the most devastating fungal disease of yam (*Dioscorea* spp.), can be produced by genomics‐assisted breeding, high‐throughput phenotyping, genetic engineering, and CRISPR/Cas9 genome editing.

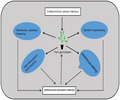

## 1 INTRODUCTION

Yam is an economically important, starchy staple for millions of people in tropical and subtropical regions of the world. Presently, West Africa is responsible for approximately 95% of total global yam production (FAOSTAT, 2018). The genus *Dioscorea* has over 600 species, but only 11 are cultivated as food crops, which are *Dioscorea alata* (greater/water/winged yam; South‐east Asia, Melanesia), *Dioscorea bulbifera* (aerial/bulbil‐bearing yam; South America, Africa, Asia, and Melanesia), *Dioscorea cayenensis* (yellow Guinea yam; West Africa), *Dioscorea dumetorum* (sweet yam; West Africa), *Dioscorea esculenta* (lesser/Asiatic yam; South‐east Asia, Melanesia), *Dioscorea japonica* (glutinous/Japanese yam; Japan), *Dioscorea nummularia* (Pacific/spiny yam; Melanesia), *Dioscorea oppositifolia* (Chinese yam; China), *Dioscorea pentaphylla* (five‐leaved yam; South‐east Asia, Melanesia), *Dioscorea rotundata* (white Guinea yam; West Africa), and *Dioscorea trifida* (aja, aje, cush‐cush, yampi; South America). However, some of the wild yam species are also important during food scarcity and are used for medicinal purposes (Govaerts et al., 2007; Tamiru et al., 2017; Verter & Vera Becvarova, 2015). Yam is rich in carbohydrate and thus provides a good source of energy. It also has unique medicinal properties on account of its rich alkaloid content and steroidal compounds (Bantilan, 2019; Mignouna et al., 2008). Some yam species are also used for the production of industrial starch.

Yam is the world's fourth most important tuber crop, after cassava, potato, and sweet potato (IITA, 2013). For example, yam is grown in several countries in Central, East, and West Africa. The yam belt region of West Africa, including Benin, Ghana, Ivory Coast, Nigeria, and Togo, is responsible for about 95% of the 72.6 million tonnes of global yam production (FAOSTAT, 2018). Nigeria is presently the lead producer, contributing over 65% of global yam production (FAOSTAT, 2018). Although different types of yams are grown globally, farmers in West Africa grow mainly *D*. *rotundata*, *D*. *alata*, and *D*. *dumetorum*.

Anthracnose or dieback disease of yam, which is caused predominantly by *Colletotrichum alatae* (Weir et al., 2012), is the most widespread of all field diseases of yam, constituting a critical problem in all yam‐producing areas of the world (Amusa et al., 2003). The effect is most severe during the rainy season. Young yam plants are highly vulnerable, but infection can occur at all growth stages in susceptible yam genotypes and progress to severe disease, causing serious yield losses (Nwadili et al., 2017). Plants can be killed without producing any tubers, or each shoot may produce several small tubers before it dies if the disease strikes early. Tuber yield losses of up to 90% have been reported in susceptible genotypes in West Africa (Akem, 1999; Mignucci et al., 1988). For example, up to 95% of farmers in Ghana reported poor yield in *D*. *alata* due to anthracnose disease. This poor yield negatively impacted their livelihoods as the farmers were unable to pay children's school fees and medical bills, service loans or purchase assets (Coffie, 2013). Among the cultivated species of yam, *D. alata* is the most susceptible to anthracnose (Amusa, 1997). However, contrary to the earlier reports of negligible attacks on other yam species (Mignucci et al., 1988), some reports have shown evidence of severe anthracnose in *D*. *rotundata* in West Africa (Abang et al., 2003; Akem, 1999; Azeteh et al., 2019).

Combating the effects of this disease through application of fungicides is detrimental to the environment; it also adds to the already high production cost and could induce development of resistant strains of the pathogen (Onyeka et al., 2006). Cultural practices such as crop rotation, destruction of infected plants, regular weeding, and planting of disease‐free materials (Nwankiti & Arene, 1978) have been used to manage the disease; however, the protection offered by these methods is often insufficient and only temporary. Host plant resistance is an environmentally friendly and sustainable management strategy to control the disease (Onyeka et al., 2006) even though the transfer of desirable traits into the crop may be stalled by several factors with conventional breeding. Several breeding programmes in India, Ivory Coast, Ghana, Guadeloupe, Nigeria, and Vanuatu are making progress towards the production of an anthracnose‐resistant hybrid (Lebot et al., 2019). However, a multidisciplinary approach of traditional plant breeding, molecular breeding, and new breeding techniques would be strategically ideal for developing new improved yam varieties to handle the challenge of variable anthracnose pathogens and accompanying pathogenicity. Therefore, researchers could explore the development of improved yam varieties with broad‐spectrum and durable resistance by applying genetic engineering to complement conventional breeding. This article reviews the potential tools that could be used to tackle yam anthracnose in West Africa along with the associated problems and future prospects.

## 2 ANTHRACNOSE: CAUSAL AGENT, DISEASE SYMPTOMS, DIAGNOSIS, AND SPREAD

*Colletotrichum* is an economically important genus worldwide, causing diseases in virtually all families of plants in the temperate, tropical, and subtropical regions of the world (Bhunjun et al., 2021; Jayawardena et al., 2016b). It is rated as the eighth most important group of plant‐pathogenic fungi worldwide. Members of the genus exist basically as pathogens but they have also been reported to lead endophytic and saprobic lifestyles (Hyde et al., 2014; Jayawardena et al., 2016a). *Colletotrichum* is a complex genus with more than 1,000 described form‐species (Fokunang et al., 1995) and it is the sole member of the family Glomerellaceae (Maharachchikumbura et al., 2016).

*Colletotrichum* that infects yam is found in all yam‐growing regions and exhibits different growth characteristics. Based on the growth characteristics (growth rate, conidia, and appressorial morphology), four broad forms of *Colletotrichum* associated with yam anthracnose have been described (Abang et al., 2002). These include the slow‐growing grey (SGG), the fast‐growing grey (FGG), the fast‐growing salmon (FGS), and the fast‐growing olive (FGO) forms. Of these, SGG is known to be the most aggressive and virulent strain in terms of spread across the yam belt region, causing 100% defoliation and premature death of up to 76% of inoculated plants (Abang et al., 2003; Mignouna et al., 2001). Some studies have shown that isolates of *Colletotrichum* from diseased yam leaves are morphologically (Winch et al., 1984) and genetically (Abang et al., 2002) diverse. However, these two studies used a broad species concept to group all isolates sourced from yam under the single name *C*. *gloeosporioides*. Weir et al. (2012) found that a yam anthracnose isolate from Nigeria together with those from Barbados (SAS8 and SAS9), Guadeloupe (cgA13, GenBank accession GQ495617), and India (CBS 304.67, GenBank accession FJ940734) belonged to the same clade and matched the SGG group described by Abang et al. (2002), and hence they were described as a distinct species named *C*. *alatae* (Weir et al., 2012). In July 2016, anthracnose‐like lesions were observed on the leaves of *D*. *alata* cultivar Da56 at a plantation in Danzhou City, Hainan Province, China. Morphological and molecular characteristics of the isolates matched descriptions of the SGG group observed in yam in West Africa (Lin et al., 2018). This isolate was also referred to as *C. alatae* (Lin et al., 2018). While several authors (Bhunjun et al., 2021; Jayawardena et al., 2016c; Lin et al., 2018; Weir et al., 2012) have adopted the name *C*. *alatae* as causal agent of yam anthracnose, some authors (Kwodaga et al., 2020) have not picked up the name and are still using the name *C*. *gloeosporioides*. These discrepancies may be due to the broad species concept of classifying all the forms into a single group. However, because SGG is the most virulent form of anthracnose infecting yam in West Africa and several other regions, with no isolates from other hosts found in the same clade (Weir et al., 2012), and based on recent reviews and studies of the genus (Bhunjun et al., 2021; Jayawardena et al., 2016c; Lin et al., 2018; Weir et al., 2012), in this work, the name *C*. *alatae* is adopted as the causal agent of yam anthracnose.

**FIGURE 1:**
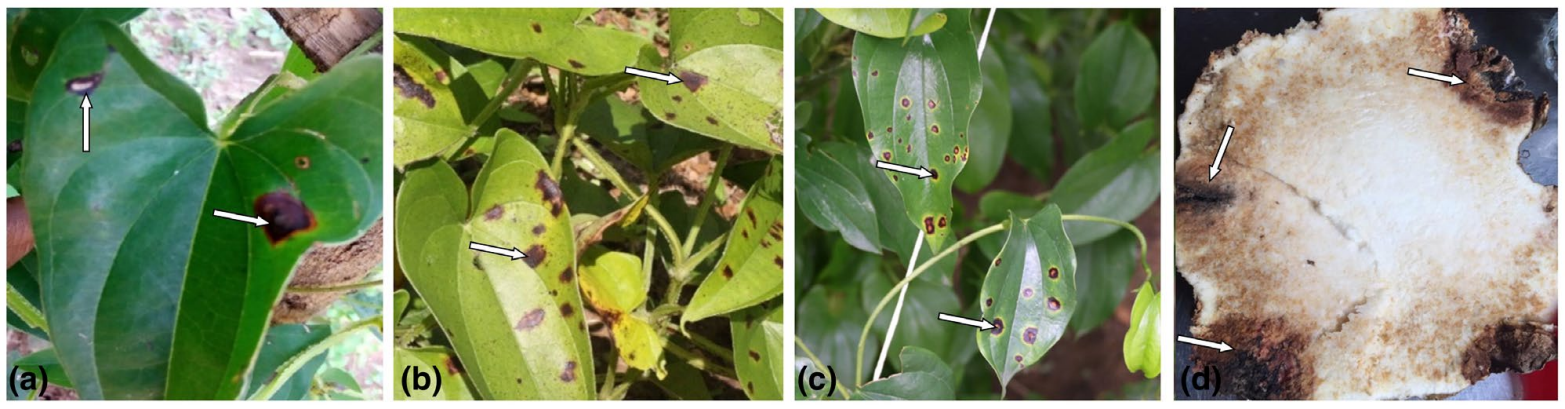
Symptoms of anthracnose in different yam tissues. (a–c) Symptoms in leaves. (d) Symptoms in yam tubers. (a) Pale yellow margins surrounding the lesions. (b,c) Dark brown spot dotting the leaf lamina. (d) Dark brown lesions on tubers. Arrows indicate the lesions

*C. alatae* attacks all parts of the yam plant, including leaves, stems, tubers, and seeds, at all stages of plant development (Abang et al., 2002) and it is present in the yam belt region and other yam‐producing countries in Africa. *C*. *alatae* begins its life cycle from germination of conidia on the plant surface (leaf, stem, or tuber) forming melanized infection structures called appressoria. This is followed by penetration of host tissue. Infection hyphae are formed in primary infected cells. This is the biotrophic stage of infection, where no symptom is visible. The necrotrophic phase of infection follows, which is characterized by the formation of thin secondary hyphae that spring from the primary hyphae and start colonizing the nearby cells. The process brings about development of visible lesions at the site of infection. Finally, conidia are formed on the surface of infected tissue and then they are dispersed by air currents, water splash, and/or insects to start another infection cycle (De Silva et al., 2019; Sharma & Kulshrestha, 2015). Symptoms start as pinpoint lesions of less than 2 mm on young yam leaves, frequently seen on the adaxial surfaces. These lesions are dark brown or black on leaves, petioles, and stems, and are usually surrounded by a chlorotic halo, which enlarges, coalesces, and eventually produces leaf necrosis and stem dieback, with withered leaves and a scorched appearance (Figure 1). Defoliation occurs in severe cases, leaving behind naked scorched vines. Death of plant cells due to toxic substances produced by the fungus can permit other pathogens to colonize (Egesi et al., 2007). Some cultivars are reported to manifest leaf chlorosis and stunted growth, while others display leaf twists or folds leading to stunted growth; these symptoms were credited to toxins from the pathogen (Nwankiti & Ene, 1984). Depending on the symptoms on infected plants, anthracnose is labelled with various names, such as dieback, scorch, canker, Apollo, blight, and anthracnose/blotch (Green & Simons, 1998; Winch et al., 1984). Anthracnose can be spread through dispersal of conidia by wind, rain, insects, and garden tools, but it is mostly spread through rain splashes of the soil containing spores on the plants. Anthracnose commences when the pathogen penetrates natural openings such as stomata and intact cuticle on the leaf surface (Nwankiti et al., 1987). Several authors (e.g., De Silva et al., 2017; Kolattukudy et al., 2000) have worked on the postinfection colonization strategies adopted by *Colletotrichum* species on fruits. However, little is known about the mechanism employed by the pathogen in colonizing yam leaf tissues. In the intracellular hemibiotrophic phase, the pathogen produces primary hyphae and infection vesicles invading the epidermal and mesophyll cells (De Silva et al., 2017). This early symptomless (biotrophic) phase of infection has been reported in anthracnose infection of yam (Jayawardena et al., 2016). This symptomless stage is followed by the secretion of cell wall‐degrading enzymes that kill the host cells. Enzymes involved in the degradation of cell walls and maceration of yam leaf tissues have been shown to enhance yam leaf tissue invasion by the pathogen (Amusa et al., 1993), besides phytotoxic secondary metabolites (pathotoxins) implicated in further cellular destruction. The virulence of yam anthracnose pathogens is also anchored on the ease of recombination of its virulence alleles and its high potential for gene flow leading to genetic diversity between geographically distant populations. These potent genetic characters enhance its rapid evolution and aid its ability to influence disease severity in a host cultivar (Abang et al., 2003).

The persistence of yam anthracnose in the farm is perpetuated by potent sources of inoculum. The most important sources of *C*. *alatae* inoculum are infested crop debris, infected tubers, and alternate hosts (Amusa, 2000; Rojas et al., 2010). It has been shown that the pathogen is capable of being transmitted from foliage to tuber, and from tuber to foliage the following season. Green (1994) has also reported that the pathogen overwinters in leaves, stems, and seeds. Weeds, such as *Acalypha ciliata*, *Calapogonium mucunoides*, *Chromolaena odorata*, *Commelina* spp., *Euphobia heterophylla*, *Ipomoea triloba*, and *Spigelia anthelmia*, have also been implicated as alternate hosts harbouring the pathogen populations of epidemic proportion with cross‐infection potential (Alleyne, 2001; Amusa, 2000). The probable spread of anthracnose through spores has been suggested through rain splash (Milgroom, 2003).

Anthracnose can be diagnosed easily by the morphological appearance of the disease on the tissues. Disease severity on leaves, lesion size, spore production on whole plants, and a pathogenicity test have been used as means to diagnose the disease. These methods have shortcomings because according to Serra et al. (2011), different species of *Colletotrichum* are capable of infecting a single host, making it difficult to differentiate in terms of their symptoms and cultural morphology (Shi et al., 2008). Molecular techniques such as PCR using species‐specific internal transcribed spacers (ITSs) (ITS1 and ITS2) have been described as the best diagnostic tool for building phylogenetic relationships in the *Colletotrichum* genus at the species level (Chagas et al., 2017; Kwodaga et al., 2020; Serra et al., 2011). Cloning and sequencing of PCR products, homology searches based on the available sequences in NCBI (Nwadili et al., 2017), information from multilocus DNA analyses, and metabolic, proteomic, morphological, physiological, biochemical, ecological, biogeographical, and mating data (Aime et al., 2021) will allow the identification of pathogens at the species level.

## 3 CONTROL AND MANAGEMENT OF YAM ANTHRACNOSE

Cultural practices are geared towards the production of healthy plants. Cultural practice is affordable and sustainable because it inhibits the buildup of disease pathogens in the soil and plants. The preventive measures usually have a long‐term effect when compared to other methods including the use of pesticides (OISAT, 2020). To control yam anthracnose, disease avoidance is key. Practices that encourage disease avoidance include early planting, removal of plants that are alternative hosts for *C. alatae*, field sanitation, planting of healthy seed yams, intercropping with barrier plants, early staking, adopting crop rotation (Jackson et al., 2000), and monitoring/screening (Infonet Biovision). Although it has been reported that the fungus can survive in an alternative host epiphytically (de Silva et al., 2021), the host range of *C*. *alatae* needs to be determined as this is an important area open to investigation. Early planting allows for quick plant canopy establishment before the months when rainfall, which favours disease proliferation, becomes severe (Egesi et al., 2007; Green, 1994).

The control of yam anthracnose has been accomplished mainly with chemical fungicides such as benomyl (benlate), maneb, chlorothalonil, and mancozeb, which require biweekly or monthly applications. However, this could damage the environment, and its frequent use could lead to the development of fungicide‐resistant strains (Onyeka et al., 2006). Fungicides can exert deleterious effects, which can delay the onset of epidemics but cannot prevent them from developing during the rainy season. In general, copper fungicides in combination with other fungicides such as mancozeb have been shown to be very effective against fungal pathogens (Johnson & Hofman, 2009), but copper alone is less effective under high disease pressure and it is phytotoxic. However, it has been reported that dithiocarbamet, mancozeb, and febran provide excellent anthracnose control in the field (Akem, 2006). Thus, treating yam tubers with fungicides such as benlate and captan has been reported to be effective in reducing fungal yam rot (Ogundana, 1971, 1981). Benomyl was very efficient and applied only twice during the growth period but its application has been discontinued. The use of tecto (thiabendazole) (Amusa & Ayinla, 1997; Ogundana, 1971, 1981), locally made dry gin (Akinnusi et al., 1987; Ogali et al., 1991), or wood ash before storage (Osai, 1993), which are known to cause little or no mammalian toxicity, has also been recommended.

Nwankiti et al. (1987) reported that the most effective and desirable means of controlling field yam diseases is by planting resistant cultivars. Resistant yam cultivars could form the basis of sustainable management strategies for anthracnose. Ongoing efforts aimed at developing high‐yielding anthracnose‐resistant yam cultivars through classical breeding have been slow considering the biological constraints related to the heterozygous and vegetative propagation of the crop. Despite its biological constraints, some progress has been reported in the development of anthracnose‐resistant hybrids through conventional breeding (Lebot et al., 2019).

Earlier investigations into the genetic control of the inheritance of anthracnose in water yam showed that resistance is likely to be dominant and quantitatively inherited (Abang et al., 2001; Petro et al., 2011). Mignouna et al. (2001), using amplified fragment length polymorphism (AFLP) markers, demonstrated that a single major dominant locus, designated as *Dcg‐1*, controls resistance in the breeding line TDa 95/00328 and that this resistance is isolate‐specific. Subsequently, some yam cultivars resistant to anthracnose have been identified. For example, TDa 1425 and TDr 2040 yam accessions in the collection of the Genetic Resource Unit of the International Institute for Tropical Agriculture (IITA) were found to be resistant to two isolates of the pathogen and were recommended for use in areas endemic with yam anthracnose (Popoola et al., 2013). Also, *D*. *alata* lines Da. 110, Jas 2, and TCR 142 were found to be highly resistant to anthracnose following laboratory and field screening in India (Arya et al., 2019). These resistant lines were recommended for further evaluation and use in breeding programmes. Resistance to yam anthracnose reported in some yam cultivars by various authors was reported to be isolate‐specific. Hopefully, a combination of both conventional and molecular techniques will be a better approach to develop yam cultivars with a wide range of stable resistance genes to protect against a broad spectrum of fungal pathogens for yam improvement.

## 4 APPLICATION OF GENOMICS‐ASSISTED BREEDING AND HIGH‐THROUGHPUT PHENOTYPING FOR CONTROL OF YAM ANTHRACNOSE

In recent years, genomics‐assisted breeding (GAB), which integrates genomic tools with high‐throughput phenotyping to improve crops, has become a powerful plant breeding strategy. GAB uses molecular markers to facilitate the prediction of phenotype from a genotype and allows breeders to select outstanding genotypes for further evaluation. Molecular markers have been used to identify and locate genes and quantitative trait loci (QTLs) linked to disease resistance in several plants. The first trait mapping on disease resistance in yam was done by Mignouna et al. (2002). The authors used a bulked segregant analysis to detect rapid amplified polymorphic DNA (RAPD) markers linked to yam mosaic virus (YMV) disease resistance in an F_1_ progeny resulting from a cross between a resistant male parent (TDr89/0144) and a susceptible female parent (TDr87/00571). They identified a single locus linked to YMV resistance and named it *Ymv‐1*. Also, they developed two RAPD markers closely linked to *Ymv‐1* in the same linkage group. These markers were successfully used to identify *Ymv‐1* resistance in *D*. *rotundata* varieties and F_1_ progeny, indicating their possible utility in marker‐assisted selection. Furthermore, Mignouna et al. (2002) used QTL mapping to identify one AFLP marker, E‐14/M52‐307, positioned on linkage group 2, that was associated with anthracnose disease resistance.

Petro et al. (2011) constructed an intraspecific genetic linkage map of *D*. *alata* using 523 polymorphic AFLP markers and nine putative QTLs on five linkage groups known for anthracnose resistance. They noted that the phenotypic variance for each QTL was between 7.0% and 32.9%, and depending on the isolate and the variable considered, the significant QTLs accounted for 26.4% to 73.7% of total phenotypic variance.

Recently, Bhattacharjee et al. (2018) developed a genetic linkage map from 380 expressed sequence tag‐simple sequence repeats (EST‐SSRs) on 20 linkage groups in order to identify QTLs controlling anthracnose resistance in *D*. *alata*. Linkage analysis conducted independently on data collected for 3 years by inoculating the mapping population with the most virulent strain of the pathogen from West Africa consistently found one QTL on linkage group 14. This QTL, found at a position interval of 71.1–84.8 cM, accounted for 68.5% of the total phenotypic variation in the average score data. This discovery could be useful in marker‐assisted breeding in developing resistance to anthracnose in *D*. *alata*.

SSR markers, due to their codominant nature, high level of polymorphism, and high abundance, have been used to identify anthracnose‐resistant and ‐susceptible *D*. *alata* genotypes (Darkwa et al., 2020). Saski et al. (2015) developed 1,152 EST‐SSRs from EST sequences generated from susceptible and resistant *D*. *alata* genotypes. In total, 388 of the EST‐SSRs showed a polymorphism rate of 34% for anthracnose on two different parental genotypes, indicating the possibility of using SSR to track anthracnose. Furthermore, they used genotyping by sequencing (GBS) tools such as EST sequencing, de novo sequencing, and GBS profiles and developed a comprehensive set of EST‐SSRs, genomic SSRs, whole‐genome single nucleotide polymorphisms (SNPs), and reduced representation SNPs for resistance to yam anthracnose in two *D*. *alata* genotypes, TDa95/00328 (resistant to anthracnose) and TDa95/310 (susceptible to anthracnose). However, the setback of the study is that many of the SNPs identified may be associated with broad‐spectrum resistance or the infection response (Darkwa et al., 2020).

Narina et al. (2011) identified genes differentially expressed in response to pathogen infection through a comparative transcriptomic analysis of infected susceptible (TDa95/0310) and two resistant yam genotypes (TDa87/01091 and TDa95/0328). They generated 15,984 ESTs in TDa95/0310; 15,196 ESTs in TDa95/0328; and 13,577 ESTs in TDa87/01091, with average sequence lengths of 411, 426, and 524 bases, respectively. TDa95/0328 and TDa87/01091 had 115 and 180 highly expressed ESTs, respectively, which were found to be linked to carbohydrate metabolism, cell wall biogenesis, lipid and amino acid metabolism, secondary and hormone metabolism, transcription factors, protein synthesis, and signalling proteins as well as multiple pathogenesis‐related and host defence‐related genes (Darkwa et al., 2020). The highly expressed ESTs in the resistant genotypes could be responsible for their tolerance to the pathogen. The limitation of this study is that the identified SNP markers may also be associated with other diseases and not anthracnose infection alone.

Screening of yam plants for anthracnose resistance, selecting and segregating infected plants will reduce the spread of the disease. Considerable advances have been made in the development of tools for the screening and detection of anthracnose. The IITA, Ibadan, Nigeria, through the AfricaYam project (https://africayam.org/the‐africayam‐new‐app‐for‐yam‐anthracnose‐disease) has developed and standardized a detached leaf assay (DLA), the “Leaf Doctor” and “ESTIMATE,” for high‐throughput screening of *D*. *alata* for anthracnose resistance (Kolade et al., 2018). The “ESTIMATE” application uses yam anthracnose standard area diagrams for image‐based phenotyping in the field and DLA. The “Leaf Doctor” and “ESTIMATE” use artificial intelligence and machine learning to accurately determine the percentage of leaf area affected by the disease (Pethybridge & Nelson, 2015). This greatly enhances the selection of promising lines for further evaluation (Kolade et al., 2018). This application is presently being adopted in different countries to rapidly phenotype yam for anthracnose resistance (Darkwa et al., 2020).

## 5 FUTURE PROSPECTS OF APPLYING GENETIC ENGINEERING STRATEGIES FOR CONTROL OF YAM ANTHRACNOSE

The use of conventional breeding to produce anthracnose‐resistant yam hybrids has shown some success. However, traditional breeding has not been exploited fully due to factors related to plant biology and low genetic variability. To overcome these hurdles, conventional breeding needs to be complemented with genetic engineering, including transgenic or genetic modification and genome editing techniques towards the improvement of the yam crop. These techniques can be used to manipulate the different genotypes of yam without any barrier to produce improved varieties with broad‐spectrum resistance to anthracnose. The genetic engineering techniques allow altering the host‐plant genome either by manipulating the endogenous genes or by overexpressing transgenes from the same or different plant species or from microbes to confer resistance to fungal pathogens. Genes or molecules involved in defence signalling, defence regulation, or other processes can easily be upregulated by transgenesis, enhancing resistance to anthracnose. The susceptibility (S) genes, which function to facilitate anthracnose infection and colonization, can be silenced, resulting in enhanced resistance to the pathogen. Through genetic engineering, essential anthracnose genes can also be silenced through RNA interference (RNAi), resulting in reduced disease incidence.

### 5.1 Transgenic approaches through overexpression of fungal resistance genes

Several studies have developed systems for transient and stable gene expression in yam, including particle bombardment (Tör et al., 1993), polyethylene glycol (PEG)‐mediated transfection of protoplasts (Tör et al., 1998), and *Agrobacterium*‐mediated transformation (Nyaboga et al., 2014; Quain et al., 2011). Among these protocols, *Agrobacterium*‐mediated transformation is the most preferred because it is easily available, facilitates the integration of large nucleotide segments with negligible rearrangements, allows for the transfer of only a single copy of the gene, and is relatively cheap. A protocol for *Agrobacterium*‐mediated genetic transformation of yam has been documented (Nyaboga et al., 2014). The authors transformed two cultivars of *D*. *rotundata* with *Agrobacterium tumefaciens* harbouring binary vectors containing *gus* and *gfp* as reporter genes and obtained a transformation efficiency ranging from 9.4% to 18.2%, depending on the cultivars, selectable marker genes, and the *Agrobacterium* strain used for transformation. This protocol could be used to overexpress transgenes for anthracnose resistance. Although no work has documented the expression of foreign genes in yam for fungal resistance, several genes conferring resistance to fungal diseases have been identified and expressed in other crops. For example, transgenic grapevine lines overexpressing a *Vitis vinifera NPR1.1* gene developed via *Agrobacterium*‐mediated transformation were reported to be resistant against powdery mildew (Le et al., 2011). Also, transgenic tomato lines overexpressing a wheat *chitinase* gene, *chi194*, under the control of the maize ubiquitin 1 promoter were found to be highly resistant to Fusarium wilt disease of tomato caused by *Fusarium oxysporum* f. sp. *lycopersici* (Girhepuje & Shinde, 2011). Mishra et al. (2016) demonstrated the control of guava wilt disease, caused by the soilborne fungus *F. oxysporum* f. sp. *psidii*, by expressing a *Trichoderma endochitinase* gene in transgenic guava (*Psidium guajava*). As revealed by in vitro pathogen inhibition assays and spore germination assays, the crude extract of the transformed plants inhibited the germination of fungal conidia and plants were resistant to wilt disease. Expression of the wasabi *defensin* gene in melon via *Agrobacterium* transformation conferred resistance to Fusarium wilt and Alternaria leaf spot (Ntui et al., 2010). In a similar way, yam could be transformed with these genes or others used in different works to confer resistance to anthracnose disease. Also, the overexpression of *Dcg‐1* in susceptible lines of yams can ultimately lead to the production of yam cultivars resistant to anthracnose. This will boost efforts aimed at producing anthracnose‐resistant varieties of yam.

### 5.2 RNAi strategy for engineering resistance against anthracnose

For several fungal diseases, including anthracnose, Botrytis rots, downy and powdery mildews, and Fusarium wilts and rots, RNAi could be seen as a promising alternative to multiple control strategies, including the use of fungicides, cultural practices, and the deployment of resistant plant cultivars. To date, the feasibility of RNAi for targeted gene silencing via the exogenous addition of synthetic double‐stranded small interfering RNAs (siRNAs) targeting specific genes has been succinctly demonstrated in several fungi. For example, a considerable advance could be made using RNAi technology in the fight against anthracnose that is caused by several genera of ascomycete fungi including *Colletotrichum lindemuthianum*, which adversely affects the yield of *Phaseolus vulgaris* (de Lima Castro et al., 2017; LeClair et al., 2015), *Colletotrichum sublineola* affecting sorghum, *Colletotrichum gloeosporioides* affecting chilli and tomato (Mahto et al., 2020), and *C*. *alatae* causing yam dieback and being the most serious disease affecting *Dioscorea* species (Figure 1), especially under intensive cultivation in the tropics (Ripoche et al., 2008). RNAi posttranscriptional gene silencing can be programmed with 21–25‐nucleotide duplexes of siRNAs and long double‐stranded RNAs (dsRNAs) corresponding to different sequences in order to induce an effective antifungal response against the replication of the fungal pathogen, both in in vitro cultures as well as during infection in the field (Machado et al., 2018; Wani et al., 2010). It is possible that, as observed naturally in many eukaryote kingdoms (Baulcombe, 2015), for example in the nematode *Caenorhabditis elegans* (Fire et al., 1998), following the microinjection of 500–700‐nucleotide siRNAs, RNAi may function as an adaptive, nucleic acid‐based defence system in anthracnose‐susceptible crop species by either inhibiting replication at different stages in the life cycle of the particular *Colletotrichum* pathogen, or simply by acting as a retrotransposon silencing mechanism. However, irrespective of the exact gene silencing pathway(s) in use, there are several proofs of principle regarding the feasibility of RNAi in the downregulation of specific genes in fungi (Li et al., 2010) including *Neurospora crassa* (Fulci & Macino, 2007; Romano & Macino, 1992), *Mucor circinelloides* (Billmyre et al., 2013), and *Saccharomyces cerevisiae* (Billmyre et al., 2013; Trieu et al., 2015).

Usually, RNAi is initiated following the entry of a long dsRNA such as an introduced transgene, a rogue genetic element, or a microbial intruder into the cell, which triggers the RNAi pathway and results in the recruitment of the enzyme Dicer. Thereafter, the Dicer enzyme cleaves the dsRNA into 21–25‐nucleotide siRNA duplexes. Subsequently, an RNA‐induced silencing complex (RISC) then distinguishes between the two siRNA strands as either sense or antisense strands. While the sense strand is degraded, the antisense strand is loaded into the RISC, which is used as guide to target mRNAs in a sequence‐specific manner. Messenger RNAs, which code for proteins, are then cleaved by the RISC. The activated RISC can repeatedly participate in mRNA degradation, thus inhibiting protein synthesis in the cell. On account of this posttranscriptional gene silencing mechanism, it could be speculated that specific dsRNAs might serve as promising vehicles that can be developed into molecular tools for genetic improvement of food crops against fungal infections, including anthracnose disease in yam.

Based on reports, as might be expected, RNAi is astounding, having the power to overwhelm fungal pathogens by turning off or silencing harmful genes (Almeida & Allshire, 2005; de Bakker et al., 2002). This is best illustrated by data from several recent investigations. It has also been reported that barley and wheat plants could be transgenically engineered to express dsRNAs that target transcripts of the virulence factor Avra10 in the fungus *Blumeria graminis*, which resulted in reduced powdery mildew infections in the crops. Furthermore, it has been demonstrated in numerous studies that host immune gene silencing is an effective approach in the control of a wide range of taxonomically unrelated filamentous fungal and oomycete pathogens (Machado et al., 2018). Aside from these, several studies using long, corresponding fragments of dsRNA also showed that fungal replication can be inhibited in both *Botrytis cinerea* and *Fusarium graminearum* (Koch et al., 2013; Wang et al., 2016).

Of paramount significance and fascination in these studies was the finding that the response, ascribed to posttranscriptional silencing of specific genes, showed exogenous transfected siRNAs and long dsRNAs that direct the sequence‐specific degradation of mRNAs encoding cognate receptors through which the fungal cells gain entry into plant cells (Machado et al., 2018). Collectively, these observations are highly encouraging and could be of immense significance in the control of fungal diseases, including anthracnose. They illustrate the fact that using intracellular RNA‐based therapeutics, developed against the right target gene(s) such as the fungal Dicer‐like 1 (*DCL1*) and *DCL2* genes (Wang et al., 2016), RNAi might serve as an effective strategy for developing a durable therapy against anthracnose in food crops including yam species. It is likely that additional successes will come from a more detailed understanding of the practical implications of using RNA‐based therapeutics through the production of dsRNA nucleotide intermediates against some prime target genes that can be used as therapy against different fungal diseases, especially anthracnose, which can be considered as a paradigm shift in crop protection.

### 5.3 CRISPR/Cas9 gene editing strategy to develop anthracnose resistance

Recent advances in genome editing technologies using site‐directed nucleases (SDNs), such as meganucleases, encoded by mobile genetic elements or introns, zinc‐finger nucleases derived from eukaryotic Cys_2_His_2_ zinc finger proteins covalently linked to the nuclease domain of the type IIS restriction enzyme *Fok*1, transcription activator‐like effector nucleases (TALENs) from TALEs of bacteria *Xanthomonas* linked with the *Fok*1 nuclease domain, and CRISPR/Cas from the adaptive immunity system of *Streptococcus pyogenes* have enabled plant scientists to manipulate desired genes in crop plants (Tripathi et al., 2020).

**FIGURE 2:**
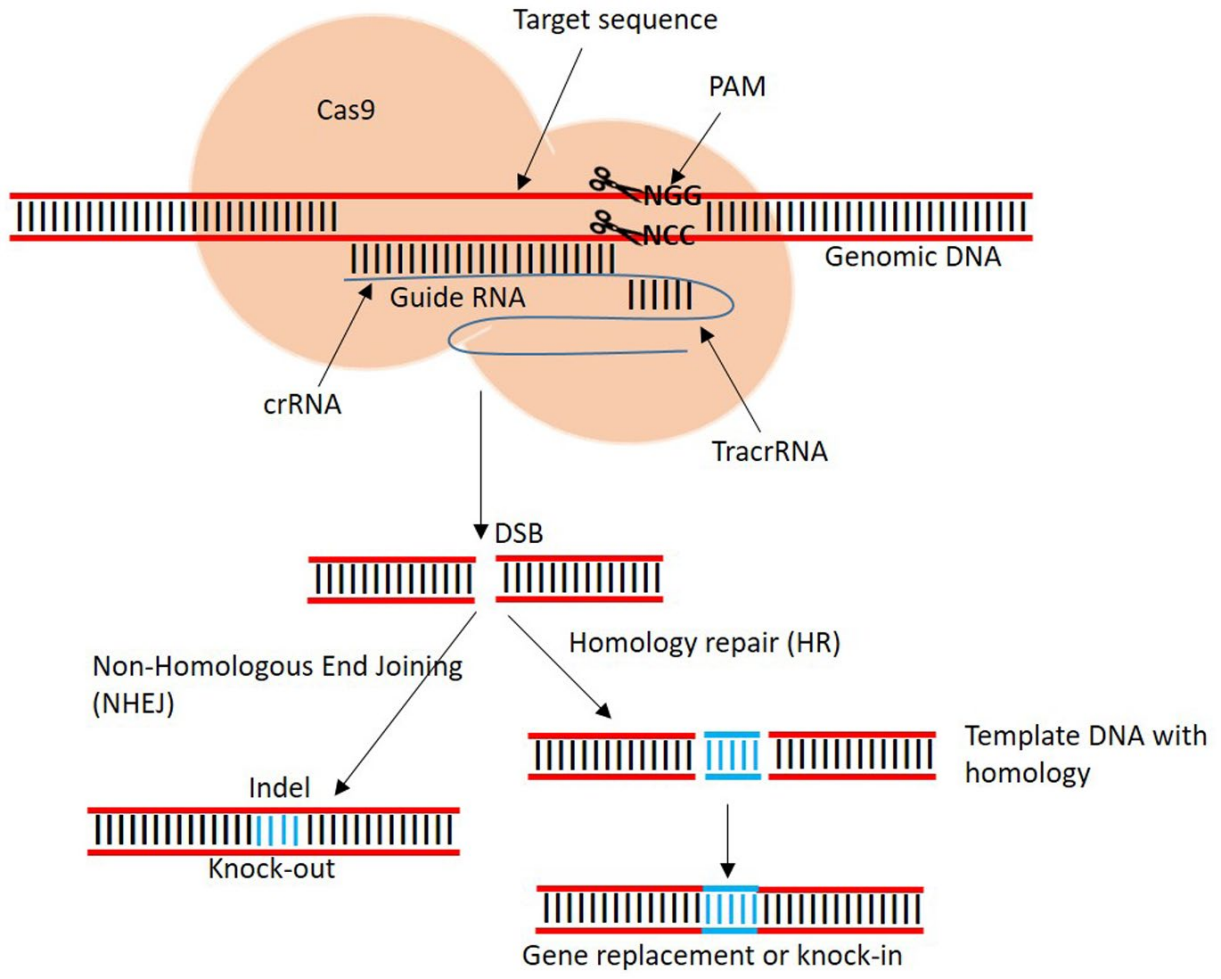
Schematic representation of the CRISPR/Cas9 gene editing mechanism. gRNA directs Cas9 to cleave the target sequence upstream of the protospacer adjacent motif (PAM), producing a double‐stranded break (DSB). The DSB is subsequently repaired either by nonhomologous end‐joining (NHEJ) or by homology‐directed repair (HDR). Repair via NHEJ produces indels (knockout), whereas repair through HDR results in knockin

Among these nucleases, CRISPR/Cas9 is the most powerful and versatile tool for crop genome editing because of its simplicity, design flexibility, and high efficiency and its ability to simultaneously edit multiple genes (Ntui et al., 2020; Tripathi et al., 2019). The CRISPR/Cas9 editing system consists of two basic components: the guide RNA (gRNA) and the Cas9 nuclease (Figure 2). Cas9 exhibits nuclease activity, recognizing target DNA by gRNA–DNA pairing between the 5′ leading sequence of gRNA. It also recognizes the protospacer adjacent motif (PAM) sequence and starts editing upstream of the sequence. The PAM is a trinucleotide sequence, usually NGG or NAG (Figure 2), where N can be any nucleotide, and serves as a recognition segment for Cas9 to start editing upstream. The gRNA consists of a scaffold and a user‐defined spacer sequence (c.20 nucleotides) for genomic sequence targeting. It directs the Cas9 to induce precise double‐stranded breaks (DSBs), which are repaired either through the nonhomologous end‐joining (NHEJ) DNA repair pathway at the target site or homology‐directed repair (HDR), resulting in small insertions/deletions (indels) or substitution of nucleotides.

Based on the type of repair, the editing can be performed by SDN1, SDN2, or SDN3 (Modrzejewski et al., 2019). SDN1 is based on NHEJ resulting in random mutations in the host genome, causing gene silencing, gene knockout, or alteration in the gene function. In SDN2, a repair template identical to the DSB is added. The DSB is then repaired via HDR, resulting in nucleotide substitution or targeted indels. In SDN3, the DSB is repaired via HDR using a repair template, which is longer than the homologous sequences in which the DSB is made, leading to the targeted insertion of foreign genes.

The CRISPR/Cas system has become the method of choice for the control of fungal diseases in plants. So far, no work has been documented using CRISPR/Cas9 to develop resistance to anthracnose in yam; however, the system has been used to induce resistance to fungal diseases in some plants species which could be applicable to yam. Fungal resistance via CRISPR/Cas9 was mainly achieved until now by targeting susceptibility (S) genes as well as ethylene‐responsive factors (Das et al., 2019; Tripathi et al., 2019). During pathogen invasion, S genes are activated by the pathogen to facilitate pathogen growth and symptom development (Boch et al., 2014; van Schie & Takken, 2014). Editing of S genes has been reported to confer resistance to the corresponding pathogen, and in some cases even broad‐spectrum resistance (Blanvillain‐Baufumé et al., 2017; Kim et al., 2019; Olivia et al., 2019; Peng et al., 2017). For example, the mildew resistance locus O (*MLO*) is the most widely known S gene locus (Das et al., 2019). *MLO* encodes a seven‐transmembrane domain calmodulin‐binding protein located at the plasma membrane (Kim et al., 2002). Its role in susceptibility toward powdery mildew disease in monocot and dicot plants has also been confirmed (Kusch & Panstruga, 2017). Editing of *MLO* in wheat (Wang et al., 2014) and tomato (Nekrasov et al., 2017) conferred resistance to powdery mildew. The *MLO* gene, together with other S genes identified in other crops such as *enhanced disease resistance 1* (*EDR1*) (Zhang et al., 2017) and *ethylene‐responsive factor* (*ERF*) (Santillán‐Martínez et al., 2020; Wang et al., 2016) would be an excellent candidate for developing resistance to yam anthracnose and other fungal diseases.

In yam, the CRISPR/Cas9‐based genome editing has been documented targeting the *phytoene desaturase* (*PDS*) gene (Syombua et al., 2021). PDS is a key enzyme in the carotenoid biosynthesis pathway, catalysing the desaturation of phytoene into lycopene. PDS is often used as a marker to establish genome editing in plants because its disruption affects photosynthesis and gibberellin and carotenoid biosynthesis, causing albinism and dwarfing, phenotypes that are easy to see. Mutated yam plantlets generated by *Agrobacterium*‐mediated transformation showed either a partial or a complete albino phenotype, and gene knockout was confirmed by sequence analysis (Syombua et al., 2021). This protocol will facilitate genome editing of yam targeting genes involved in resistance to anthracnose as well as other fungal diseases.

## 6 CHALLENGES IN DEVELOPING ANTHRACNOSE‐RESISTANT YAM THROUGH GENETIC ENGINEERING

Management of anthracnose through cultural practices and production of anthracnose‐resistant yam varieties through conventional breeding has shown some success. GAB has been used to create some levels of resistance, but the evolution of new ecotypes of the fungus requires the exploration of new breeding techniques for developing broad‐spectrum and durable resistance in yam against fungal pathogens.

Biotechnological approaches such as overexpression of fungus resistance genes, RNAi, and genome editing require an efficient yam genetic transformation protocol. Currently, yam genetic transformation is still a bottleneck that could hinder the rapid production of anthracnose‐resistant yam cultivars in West Africa. In a recently published article on genome editing of yam (Syombua et al., 2021), only six transgenic events were recovered from several hundreds of explants cocultivated with *Agrobacterium*. This indicates that yam genetic transformation is still a major challenge. Developing an efficient yam transformation protocol, at least for the cultivars preferred by farmers, is thus a necessary requirement for improving yam, not just for anthracnose resistance but also for other agronomically important traits. Yam breeders/biotechnologists could explore the possibilities of using alternative ways to deliver transgenes to yam cells in order to bypass the laborious work of tissue culture. Methods such as agroinfiltration or in planta transformation could be adopted. Viral vector‐based platforms for rapid and efficient delivery of overexpression, RNAi, and CRISPR/Cas9 constructs could be adopted. YMV may be an excellent candidate for technologies focused on viral vectors. Like the tobacco rattle virus (TRV) vector, which has been commonly used as a vector to alter plants, YMV may be genetically engineered to bear plasmids for onward transmission to yam cells through agroinfiltration (Liu et al., 2002; Ntui et al., 2013). Much like TRV, YMV is an RNA virus, making it a useful candidate for viral‐based transformation. Dioscorea alata bacilliform virus (DaBV) is another potential virus that could be adapted to bear plasmids. DaBV is a badnavirus that is known to incorporate into the genome of the host and cause symptoms under conditions of stress. DaBV is thus an excellent candidate for modification as a vector to deliver CRISPR/Cas9, RNAi, and overexpression constructs into yam cells. The modification of yam‐based viruses as vectors could facilitate the production of anthracnose‐resistant yam by agroinfiltration.

In planta transformation should be tried to rapidly develop resistance to anthracnose. In planta transformation involving a direct transformation of plant parts has been established as an innovative and simple technique for plant transformation. Whole yam plants, shoot tips, floral parts, or female reproductive parts such as zygotes, embryos, and seeds should be exploited and optimized for in planta transformation of yam. This will overcome the challenges of transformation and the problem of tissue culture‐induced genetic variability in the transformants.

The development of yam cultivars resistant to anthracnose through overexpression of a single antimicrobial gene may result in partial resistance and, in some cases, in resistance breakdown. Therefore, pyramiding (stacking) of some genes conferring resistance to fungal diseases might play a vital role in providing long‐lasting resistance to anthracnose. Cotransformation or the use of a marker‐free approach will promote the stacking of fungus resistance genes in yam.

Successful application of CRISPR/Cas9‐based genome editing to induce resistance to anthracnose will require the availability of well‐annotated genome sequences. With the recently developed genome editing protocol for yam and the available whole‐genome sequence of *D*. *rotundata* and *D*. *alata*, S gene sequences known to confer resistance to fungal diseases in other crops could be identified and edited in yam. Multiplexing of two or more of such genes may result in durable resistance to anthracnose.

Generation of anthracnose‐resistant yam either by classical genetic engineering or genome editing will be regulated in some countries in West Africa and may reduce its acceptability. Yam is vegetatively propagated and even with CRISP/Cas9‐mediated plasmid delivery, transgenes cannot be removed by segregation as in sexually propagated crops. This will be a major limitation of using these technologies to develop anthracnose‐resistant yam genotypes. However, direct delivery of preassembled Cas9 protein–gRNA ribonucleoproteins (RNPs) into yam cells could overcome these limitations as the RNPs mutate the target sites immediately after delivery and then get rapidly degraded by endogenous proteases, leaving no traces of foreign DNA elements. The mutant genotypes will be accepted without any major regulatory issues. The use of more genomic tools will contribute to the production of anthracnose‐resistant genotypes and help to overcome the regulatory challenges of classical genetic engineering and genome editing.

## 7 CONCLUSION

Yam is an important staple food crop in West Africa and plays key roles in income generation and the sociocultural life of smallholder farmers. Anthracnose is the most important fungal disease affecting yam production and causing severe economic hardship to yam producers. The production of yam resistance to anthracnose by cultural practices and conventional breeding is a major challenge. The use of anthracnose‐resistant yam varieties is the most sustainable way of reducing losses due to this fungus. The development of durable anthracnose‐resistant varieties through conventional breeding using resistant germplasm from Asia and adoption of GAB and modern biotechnological tools will speed up the production of anthracnose‐resistant yam varieties. The development of a robust genetic transformation protocol and in planta transformation techniques, as well as agroinfiltration protocols using a viral vector‐based platform, will facilitate the production of anthracnose‐resistant yam cultivars. The availability of whole‐genome sequence information of yam will enable the identification and editing of S genes conferring resistance to fungal pathogens. These technologies, if developed, could facilitate the production of anthracnose‐resistant yam cultivars and hence increase food security and income generation for yam farmers in West Africa.

## CONFLICT OF INTEREST

The authors declare that no conflicts of interest exist.

## AUTHOR CONTRIBUTIONS

E.A.U. and E.E.E. conceived the original concept. E.A.U., V.O.N., E.E.I., A.A.M., J.N.T., N.I.O., M.O.A., J.O.P., E.A.B., L.T., and E.E.E. wrote the manuscript. V.O.N., J.O.P., E.E.I., and J.N.T. prepared the figures.

## Data Availability

Data sharing is not applicable to this article as no new data were created or analysed.
